# 46 Transfusions in Older Adults: Secondary Analysis of the Transfusion Requirement in Burn Care Evaluation Study

**DOI:** 10.1093/jbcr/iraf019.046

**Published:** 2025-04-01

**Authors:** Kanika Gulia, Jason Heard, Soman Sen, Tina Palmieri, Kathleen Romanowski

**Affiliations:** California Northstate University College of Medicine; University of California Davis; University of California, Davis and Shriners Children’s; University of California, Davis and Shriners Children’s; University of California, Davis and Shriners Children’s

## Abstract

**Introduction:**

It is common for burn patients to require blood transfusions as part of their treatment. Various transfusion strategies have been studied to manage these needs. Previous studies, including the Transfusion Requirement in Burn Care Evaluation (TRIBE) study, have examined restrictive (transfusing to a hemoglobin ≥ 7g/dL) and liberal (transfusing to a hemoglobin ≥ 10 g/dL) transfusion strategies. They found that both strategies yield similar outcomes in the general population. Older adult burn patients present unique physiological challenges, but these transfusion strategies have not been specifically evaluated in this group. This study aims to assess the impact of age and transfusion strategies on outcomes, including mortality and transfusion requirements.

**Methods:**

A secondary analysis from the prospective, randomized, multicenter TRIBE study was conducted. Patients were randomized to either a liberal or restrictive treatment strategy. The relationship between age, transfusion strategy, and outcomes was examined. Statistical analyses, including Chi-square or Fisher’s Exact test for categorical variables, Wilcoxon two-sample, or Kruskal-Wallis test for continuous variables, were conducted. Spearman’s Correlation Coefficient was used for comparing two continuous variables, and multivariable regression analysis for mortality was performed.

**Results:**

In total, 347 patients with a median age of 41 (Interquartile Range [IQR] = 26) years, 274 men, and 73 women, were analyzed. The median total body surface area burned (TBSA) was 32% (IQR = 22), 23% had inhalation injuries, and 12.4% died during the study period. Mortality was significantly associated with age (Odds Ratio [OR] 1.052, p < 0.0001), but not with liberal or restrictive transfusion strategies (p = 0.53). On multivariate logistic regression analysis, age was independently associated with mortality (OR 1.052, p < 0.0001), while transfusion strategy, controlling for age, was not associated with mortality (p = 0.34). There was no significant correlation between age and the number of total transfusions required or transfusions of each blood product type, regardless of the transfusion strategy.

**Conclusions:**

The TRIBE study data, when examined by age, showed no differences in the number or volume of transfusions required for each blood product. Additionally, transfusion strategy was not a predictor of mortality when controlling for age and burn size. Established transfusion triggers seem applicable to older adults, though further research is needed.

**Applicability of Research to Practice:**

Previously established transfusion triggers appear applicable to older adult burn patients.

**Funding for the Study:**

N/A

